# Outcome of resectable distal cholangiocarcinoma in a single-centre Western patient cohort: comparison of the 7^th^ and 8^th^ edition of the UICC/AJCC TNM classification

**DOI:** 10.2340/1651-226X.2026.44965

**Published:** 2026-02-19

**Authors:** Maia Blomhoff Holm, Sondre Busund, Rahul Rihel, Mushegh A. Sahakyan, Ivar Prydz Gladhaug, Caroline S. Verbeke, Sheraz Yaqub, Dyre Kleive

**Affiliations:** a Department of Pathology, Oslo University Hospital, Oslo, Norway; b Institute of Clinical Medicine, University of Oslo, Oslo, Norway; c Department of HPB Surgery, Rikshospitalet, Oslo University Hospital, Oslo, Norway; d The Intervention Center, Rikshospitalet, Oslo University Hospital, Norway, Oslo; e Department of Surgery, Ringerike Hospital, Vestre Viken Hospital Trust, Ringerike, Norway; f Department of Surgery N1, Yerevan State Medical University, Yerevan, Armenia

**Keywords:** Distal cholangiocarcinoma, TNM classification, depth of invasion, prognostic stratification

## Abstract

**Background and purpose:**

Distal cholangiocarcinoma (CCA) is a rare malignancy with poor prognosis, even after surgical resection. Accurate staging is essential for guiding treatment and predicting outcomes. The 8th edition of the Union for International Cancer Control (UICC)/The American Joint Committee on Cancer (AJCC) TNM classification introduced depth of tumour invasion (DOI) as the criterion for T-staging (T1–T3) and a three-tiered lymph node (N) classification. This study evaluates patient stratification and prognostic accuracy of the 8th versus 7^th^ edition in a single-centre Western cohort and discusses difficulties with measuring DOI.

**Patient/material and methods:**

Patients undergoing pancreatoduodenectomy for distal CCA at Oslo University Hospital (2015–2021) were retrospectively analysed. Tumours were restaged according to the 7^th^ and 8^th^ TNM editions. Survival was assessed using Kaplan–Meier estimates and log-rank tests to compare prognostic accuracy.

**Results:**

Seventy-one patients were included. Using the 7^th^ edition, most cancers (94.4%, 67 patients) were categorised as T3. With the 8^th^ edition, stage redistribution was notable: T2 included 45 patients (63.4%) and T3 included 22 (31.0%). Five-year survival was significantly better for T2 (31.8%) than T3 (10.5%) according to the 8th edition, demonstrating improved discrimination. The revised N classification provided better prognostic distinction, with median survival of 30 months for N1 (1–3 nodes) and 23 months for N2 (≥4 nodes).

**Interpretation:**

The 8th edition provides more accurate prognostic stratification of distal CCA compared to the 7th edition but requires meticulous, standardised pathology assessment to ensure accurate prognosis and appropriate post-surgical management.

## Introduction

Cholangiocarcinoma (CCA) is classified based on the tumour site as intrahepatic, perihilar, and distal; distal CCA accounts for approximately 30% – 40% of the cases [1, 2]. Surgery remains the primary treatment for resectable distal CCA, but even following complete tumour removal, overall survival remains poor [3]. In a recent Western cohort of distal CCA, the median overall survival was 21.9 months following surgery [4]. Systemic treatment results in limited survival benefit, and many patients do not receive adjuvant chemotherapy [4–6]. According to the National Comprehensive Cancer Network® (NCCN) guidelines, observation may be considered after margin-negative and node-negative resections. In contrast, systemic therapy is recommended for patients with margin or regional lymph node involvement [3].

Continuously striving to improve cancer staging, the TNM classification for distal CCA has undergone multiple revisions (Table 1). In 2017, the 8^th^ edition of the TNM classification introduced a new T-staging criterion based on the measurement of the depth of tumour invasion (DOI) with the following threshold: less than 5 mm (T1), 5–12 mm (T2), and > 12 mm (T3) [7, 8]. DOI thus replaced *tumour extent*, which had been the T-staging criterion in the 7^th^ edition. An important reason for this revision was the difficulty with clearly delineating the bile duct wall from the surrounding adjacent pancreas, as the extrahepatic biliary tree lacks a continuous muscle layer, and discrete tissue boundaries may be effaced by inflammatory changes or desmoplastic stromal reaction to invasive cancer [9, 10]. Furthermore, the lymph node classification was also revised in the 8^th^ edition with the introduction of distinct N-categories for 1–3 regional lymph node metastases (N1) and metastasis in ≥ 4 regional lymph nodes (N2) (Table 1). These changes aimed to improve both staging accuracy and prognostic stratification in distal CCA.

**Table 1:** Comparing 7^th^ and 8^th^ ed. UICC/AJCC TNM classification and clinical stages.

Category	7^th^ edition	8^th^ edition
**Tis**	Carcinoma *in situ*	Carcinoma *in situ*/high-grade dysplasia
**T1**	Tumour confined to the bile duct histologically	T1: DOI ≤ 5 mm
**T2**	Tumour invades beyond the wall of the bile duct	T2: DOI > 5 mm and ≤ 12
**T3**	Tumour invades the gallbladder, pancreas, duodenum, or other adjacent organs without involving the celiac axis or the superior mesenteric artery	T3: DOI > 12 mm
**T4**	Tumour involves the coeliac axis or the superior mesenteric artery	Tumour involves the celiac axis, superior mesenteric artery, and/or common hepatic artery
**N0**	N0: No regional lymph node metastasis	N0: No regional lymph node metastasis
**N1**	N1: Regional lymph node metastasis	N1: 1–3 regional lymph node metastases
**N2**	Not defined	N2: ≥ 4 regional lymph node metastases
**Stage**	7th edition	8th edition
**Stage 0**	Tis N0M0	Tis N0M0
**Stage 1**	Stage 1A: T1N0M0, Stage 1B: T2N0M0	T1N0M0
**Stage 2a**	T3N0M0	T1N1M0 or T2N0M0
**Stage 2b**	T1-3N1M0	T2N1M0 or T3N0M0 or T3N1M0
**Stage 3a**	Stage 3: T4, any N, M0	T1N2M0 or T2N2M0 or T3N2M0
**Stage 3b**		T4N0M0 or T4N1M0 or T4N2M0
**Stage 4**	Any T, any N, M1	Any T, any N, M1

Moon et al. paved the way for the new staging system, validating an alternative staging system that closely resembled the 8^th^ edition TNM classification [11]. Two subsequent studies demonstrated the improved prognostic stratification of the 8^th^ edition in Asian cohorts [12, 13]. However, one study [13] reported that DOI was unmeasurable in over 50% of cases, highlighting difficulties in pathology assessment. Indeed, little attention has been paid to the practical execution of the pathology assessment, both by the AJCC/UICC and by national pathology guidelines [14, 15]. This lack of guidance bears the risk of divergent approaches and, consequently, considerable variation among pathology departments. Similar challenges have also been noted in other aspects of pathology examination, including differentiating distal CCA from other periampullary adenocarcinomas [16, 17] and determining margin status, with reported R0 rates for distal CCA ranging from 50% to 90% [18–20]. The lack of standardised pathology assessment, combined with the fact that the updated TNM classification has not been validated in a Western population, highlights the need for further validation. Furthermore, large consensus statements show variation in incidence and mortality between Eastern and Western populations, which are largely attributed to differences in environmental exposures and potentially also to genetic variation [21].

This study aims to compare prognostic stratification and survival prediction according to the 7^th^ versus 8^th^ editions of the AJCC/UICC TNM classification in a single-centre Western cohort of patients with distal CCA. In addition, the study seeks to identify the challenges associated with measurement of DOI in pancreatoduodenectomy (PD) specimens, highlighting the need for standardised pathology assessment. The manuscript was completed in accordance with the Strengthening the Reporting of Observational Studies in Epidemiology (STROBE) guidelines [22].

## Patients/material and methods

All patients who underwent pancreatoduodenectomy for histologically confirmed distal CCA at Oslo University Hospital between January 2015 and December 2021 were eligible for inclusion. A multidisciplinary team evaluated all patients before referring them for surgery. Pancreatoduodenectomy with standard lymphadenectomy was performed [23]. No changes in perioperative management were done throughout the study period. Patients with other periampullary or pancreatic head malignancies were excluded. Eligible patients were identified from the institutional surgical database. Follow-up information was obtained from hospital medical records. All patients were followed for a minimum of 2 years, with the last follow-up performed on December 31, 2023. The local hospital data protection officer approved the study, and waived the need for informed consent (case number 2015-13400).

The primary outcome was overall survival (OS), defined as the interval between surgery and death or last follow-up. Exposures of interest included T-stage and N-stage according to the AJCC/UICC 7^th^ and 8^th^ edition TNM classifications. Additional predictors assessed included tumour size, tumour location (intra- vs. extrapancreatic bile duct), resection margin status (R0 vs. R1, with ≤ 1 mm considered involved), vascular-, lymphatic- and perineural invasion, and severe postoperative complications (Clavien–Dindo grade ≥ 3a) [24].

### Pathology assessment

All specimens had been evaluated by an experienced pancreas pathologist (C.V.), and were retrospectively reclassified according to the AJCC/UICC TNM 7^th^ (2015–2017) and 8^th^ edition (2018–2021) by two experienced pancreas pathologists (C.V., M.B.), who were blinded to the original pathology reports and each other’s reviews. All specimens were examined using the same standardised protocols for macroscopic examination and pathology reporting during the entire inclusion period. Standardised specimen dissection was based on serial axial slicing at 3 mm intervals after multicolour-coded inking of the specimens’ surfaces, as previously described [25, 26] and recommended by (inter-)national guidelines [15, 27]. The standardised protocol also included detailed photographic documentation and extensive tissue sampling, including complete embedding of the entire common bile duct (CBD), en bloc with the nearest specimen surfaces. In addition to the transection margins of the pancreatic neck, CBD, stomach, and/or duodenum, all circumferential margins (posterior, superior mesenteric vein [SMV], superior mesenteric artery [SMA]) were thoroughly evaluated based on a 1 mm clearance. Of note, the circumferential margin of the extrapancreatic CBD was also included in the evaluation (1 mm clearance). Moreover, the anterior surface was evaluated based on 0 mm clearance.

**Figure 1:**
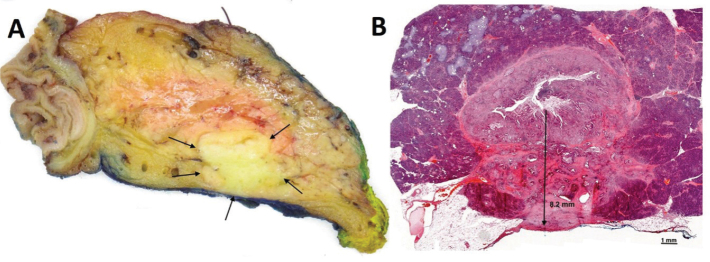
Depth of tumour invasion measured from the luminal surface of the tumour. (a) Axial slice through Whipple specimen (viewed from below, corresponding to CT view). The specimen shows carcinoma of the common bile duct with infiltration into surrounding pancreatic parenchyma(arrows), close to the posterior specimen surface (inked blue), resulting in a slit-like narrowing of the bile duct lumen. (b) H&E section showing maximum depth of tumour invasion (8.2 mm).

The DOI was assessed in all tissue sections with a proper cross-section of the CBD to identify the maximum DOI in each case. Measurements were taken from the most superficial part of the tumour to the deepest point of invasion (Figure 1).

### Statistical analysis

Continuous data are presented as means (standard deviation [SD]) or medians (range), depending on their distribution, while categorical data are presented as frequencies (percentages). Associations between study parameters and survival outcomes were analysed using the log-rank test, and Kaplan-Meier survival curves were generated. Survival was expressed as median with a 95% confidence interval (CI). A *p*-value of < 0.05 was considered statistically significant. Uni- and multivariable Cox regression analyses of prognostic factors for survival were performed. Proportional hazard assumptions were tested by checking on time dependence (time varying covariates) demonstrating the constant effect of the variables on survival over time. Data analyses were performed using Statistical Package for the Social Sciences (SPSS) software (IBM SPSS Statistics, version 18.0).

## Results

A total of 745 PDs were performed during the study period. Of these, 674 patients were excluded due to diagnoses other than distal CCA. Hence, 71 patients (9.5%) were included in the analysis. Patient demographics and perioperative details are summarised in Table 2. A preoperative biliary stent was placed in 53 patients (74.6%). Pylorus-preserving PD was the most performed surgical procedure (87.3%). Severe morbidity occurred in 29 patients (40.8%), and the 90-day mortality rate was 5.6% (4 patients).

**Table 2:** Demographics and perioperative data in patients undergoing pancreatoduodenectomy for distal bile duct cancer.

Parameters	*n* = 71
Age, years, mean (SD)	69.7 (7.8)
Gender, *n* (%)	
Female	30 (42.3%)
Male	41 (57.7%)
Body mass index, kg/m^2^, mean (SD)	25.9 (3.2)
Presence of comorbidity, *n* (%)	57 (80.3%)
Hypertension, *n* (%)	37 (52.1%)
Cardiovascular disease, (%)	19 (26.8%)
Diabetes mellitus, *n* (%)	12 (16.9%)
Total number of comorbidities, median (range)	2 (0–7)
ASA score, *n* (%)	
II	37 (52.1%)
III	34 (47.9%)
Preoperative Ca 19-9, U/mL, median (range)	74 (5–1,102)
Preoperative serum bilirubin level, µmol/L, median (range)	28 (3–469)
Preoperative biliary stent, *n* (%)	53 (74.6%)
Pylorus-preserving procedure, *n* (%)	62 (87.3%)
Vascular reconstruction, *n* (%)	6 (8.5%)
Operative time, min, mean (SD)	336 (73)
Morbidity, *n* (%)	48 (67.6%)
Severe morbidity, *n* (%)	29 (40.8%)
Reoperation, *n* (%)	17 (23.9%)
90-day mortality, *n* (%)	4 (5.6%)
Postoperative stay, days, median (range)	9 (5–86)

### Pathology findings

**Figure 2:**
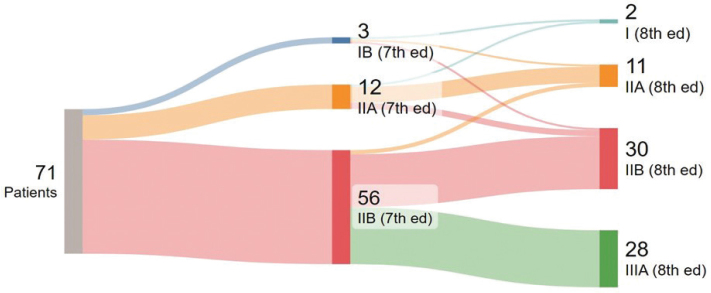
Sankey plot illustrating differences in distribution of clinical stages for TNM 7^th^ and 8^th^ classification systems.

Mean tumour size was 28.1 mm. In 34 patients (47.9%), the cancer involved both the intra- and extrapancreatic parts of the CBD. In 33 patients (46.5%), it was confined to the intrapancreatic CBD while in four patients (5.6%), the tumour was limited to the extrapancreatic portion (Table 3). Using the 7^th^ edition of TNM classification, most cancers (94.4%, 67 patients) were categorised as T3. In contrast, when applying the 8^th^ edition, T2 and T3 accounted for 45 patients (63.4%) and 22 patients (31.0%), respectively. In seven of the 71 cases (10.0%), tangential sectioning rendered measuring of DOI difficult. Lymph node involvement was frequent; according to the 7^th^ edition, 56 patients (78.9%) were classified as N1, while application of the 8^th^ edition criteria resulted in 28 patients (39.4%) being classified as N1 and 28 patients (39.4%) as N2. A high rate of microscopic margin involvement (71.8%) was observed; the three most frequently involved margins were those towards the SMV (36.6%), SMA (33.8%), and the circumferential resection margin of the extrapancreatic CBD (25.4%). Table 4 and Figure 2 illustrate redistribution between the 7^th^ and 8^th^ editions. According to the 7^th^ edition, 56 patients (78.8%) were clinical stage IIB, whereas in the 8^th^ edition, about half of these patients were shifted to clinical stage IIIA (Figure 2).

**Table 3:** Pathology findings.

Parameters	*n* = 71
Tumour localisation, *n* (%)	
Intrapancreatic	33 (46.5%)
Extrapancreatic	4 (5.6%)
Intra- and extrapancreatic	34 (47.9%)
Tumour size, mm, mean (SD)	28.1 (7.5)
T stage (7^th^ edition)	
T2	4 (5.6%)
T3	67 (94.4%)
T stage (8^th^ edition)	
T1	4 (5.6%)
T2	45 (63.4%)
T3	22 (31%)
Lymph node yield, mean (SD)	18 (6)
Positive lymph nodes, mean (SD)	3 (3)
Lymph node ratio, mean (SD)	16.6 (1.6)
N stage (7^th^ edition)	
N0	15 (21.1%)
N1	56 (78.9%)
N stage (8^th^ edition)	
N0	15 (21.1%)
N1	28 (39.4%)
N2	28 (39.4%)
Grade of differentiation, *n* (%)	
Well	8 (11.3%)
Moderate	28 (39.4%)
Poor	35 (49.3%)
Lymphatic vessel invasion, *n* (%)	59 (83.1%)
Vascular invasion, *n* (%)	39 (54.9%)
Perineural invasion, *n* (%)	66 (93%)
Margin status, *n* (%)	
R0	20 (28.2%)
R1	51 (71.8%)
Positive margins and surfaces, *n* (%)	
Anterior	2 (2.8%)
Posterior	20 (28.2%)
Pancreatic transection margin	9 (12.7%)
Duodenum/stomach	0 (0%)
Bile duct transection margin	7 (9.9%)
Bile duct circumferential margin†	18 (25.4%)
SMV	26 (36.6%)
SMA	24 (33.8%)
Venous resection margin	1 (1.4%)

**Table 4:** Differences between the 7^th^ and 8^th^ editions of TNM classification – (a) pT stage, (b) pN stage, (c) TNM stage.

(a)										
		TNM (8^th^ edition)								Total
		pT1		pT2				pT3		

TNM (7^th^ edition)	pT2	2		1				1		**4**
	pT3	2		44				21		**67**
**Total**		**4**		**45**				**22**		**71**

(b)										
		TNM (8^th^ edition)								Total
		pN0			pN1		pN2			

TNM (7^th^ edition)	pN0	15			0		0			**15**
	pN1	0			28		28			**56**
**Total**		**15**			**28**		**28**			**71**

(c)										
		TNM (8^th^ edition)								Total
		I	IIA			IIB			IIIA	

TNM (7^th^ edition)	IB	1	1			1			0	**3**
	IIA	1	8			3			0	**12**
	IIB	0	2			26			28	**56**
**Total**		**2**	**11**			**30**			**28**	**71**

### Survival for T- and N-categories and clinical stages according to 7^th^ and 8^th^ editions

Supplementary Figures S1, S2, and S3 show survival outcomes based on the 7^th^ and 8^th^ editions of the TNM classification. Survival differed significantly for patients classified as T3 with the median survival being 27 months (95% CI: 20.1–33.9) according to the 7^th^ edition compared to 23 months (95% CI: 16.6–29.4) for the 8^th^ edition (Figure S1). For N-classification, median survival was 51 months for N0, 30 months (95% CI: 18.6–41.5) for N1 and 23 months (95% CI: 19.3–26.7) for N2, with the difference between pN0 and pN2 reaching statistical significance (*p* = 0.019, Figure S2). Regarding the overall TNM-stage (Figure S3), significant differences in median survival were observed between clinical stage IIA (7^th^ edition, 32 months) and clinical stage IIIA (8^th^ edition, 23 months, *p* = 0.048). Finally, survival differed significantly also between clinical stage IIA and stage IIIA (both 8^th^ edition), with a median survival that was not reached and 23 months, respectively (*p* = 0.021). No significant survival difference was observed based on tumour location (intrapancreatic: 38 months vs. intra- and extrapancreatic: 24 months, *p* = 0.12, Figure S4). Independent predictors of poor survival included venous invasion (hazard ratio [HR] 1.84, 95% CI: 1.02–3.29, *p* = 0.042) and R1-status (HR 3.09, 95% CI: 1.37–6.92, *p* = 0.007, Table S1, supplementary material).

## Discussion and conclusion

This study investigates the outcomes of resectable distal CCA in a single-centre Western cohort, comparing the prognostic impact of the 7^th^ and 8^th^ edition of the UICC/AJCC TNM classification. Our results highlight the major advantage of the 8^th^ edition in that T-staging based on measurement of DOI resulted in a more even distribution of cancers between the categories T2 and T3, compared to the 7^th^ edition. When classified according to the 7^th^ edition, nearly all distal CCA cases in our study were assigned to category T3 (91.5% of cases). This indicates that the vast majority of distal CCA have grown beyond the bile duct wall at the time of resection, and that the extent (‘depth’) of invasion into the surrounding tissues, that is, the pancreas, is a more distinguishing feature. We believe that the more subtle microanatomical delineation between the bile duct wall and the surrounding tissues is the reason for a much higher proportion of T2 cases in other studies. In addition, 5-year survival rate for T3 tumours according to the 7^th^ edition was 24.4%, illustrating the imprecision of the 7^th^ edition in distinguishing survival outcomes among different groups. Patients with cancers classified as T2 according to the 8^th^ edition had a significantly better 5-year survival rate (31.8%) than those classified as T3 (10.5%), demonstrating how DOI is prognostically relevant. This aligns with the findings from Jun et al. [12], who reported similar significant survival differences between T-stages based on DOI, with 5-year survival rates of approximately 42.0% for T2 tumours and 12.0% for T3 tumours.

The changes in N-classification that were introduced in the 8^th^ edition also improve prognostic stratification. According to the 7^th^ edition, patients with nodal involvement were classified as N1, without further distinction reflecting the metastatic burden. In contrast, the 8^th^ edition introduced a three-tiered classification. Moon et al. [11] reported that median survival dropped from 79.2 months in N0 patients to 28.8 months N1 patients, and just 10.9 months in N2 patients. Our study yielded similar results, with median survival of 51, 30 and 23 months for N0, N1 and N2, respectively. Kang et al. [28] also support the improved prognostic accuracy of the 8^th^ edition, reporting 5-year survival rates of 54.4% for N0, 33.6% for N1, and just 4.8% for N2.

**Figure 3:**
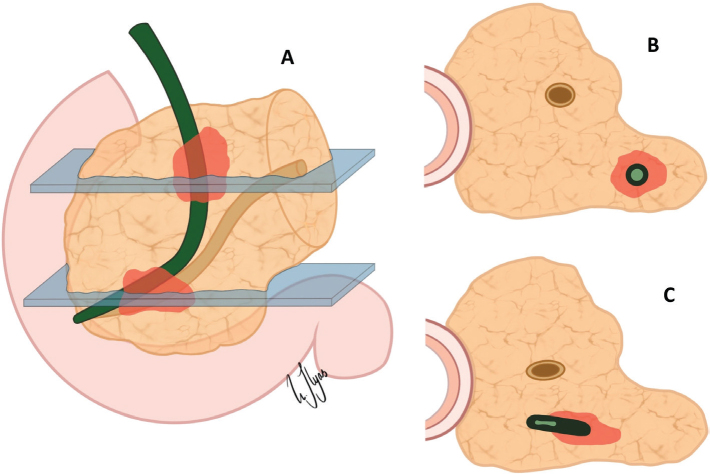
Challenges in pathology assessment – Tangential sectioning of the curved intrapancreatic bile duct. Challenges in slicing the intrapancreatic bile duct. (a) Different axial sections through the curved bile duct. (b) Proper cross-sectioning enables accurate depth of invasion (DOI) measurement. (c) Tangential sectioning deforms the lumen and may cause over- or underestimation of DOI.

The improved staging introduced by the 8^th^ edition of the TNM classification enables clinicians to better identify high-risk patients, such as those with deeply invasive tumours or extensive lymph node involvement, who may benefit from additional therapies or closer post-operative follow-up. While post-operative systemic therapy is not standard of care in some countries [4–6], the NCCN guidelines clearly state that observation only with no further treatment after surgery is an option only for patients with R0/N0 disease, whereas systemic therapy is recommended for R1 or N1/2 disease. Consequently, the clinical effectiveness of this classification depends on careful pathological assessment, in particular accurate measurement of DOI and thorough examination of the lymph nodes and margins. While the latter is extensively discussed for distal CCA and other cancers treated with PD, practical difficulties related to the measurement of DOI have not received much attention. To obtain an accurate measurement of DOI, examining full cross-sections at multiple levels through the entire length of the bile duct cancer is key. In practice, this means that the CBD should be serially sectioned in the perpendicular plane. However, as the bile duct follows a curved course and because current specimen dissection approaches are all based on parallel sectioning (either of the entire or the bivalved head of pancreas), tangential sectioning of a part of the bile duct may occur (Figure 3). If this happens at the level of the tumour, accurate measurement of the DOI may be difficult to achieve. To avoid tangential sectioning, fan-like slicing adapted to the curved shape of the intrapancreatic CBD would be needed. This is practically not feasible, however, as the course and angulation of the bile duct vary considerably between cases. The impracticability of fan-like sectioning is exemplified by Aoyama et al. who reported that their attempt at fan-like sectioning resulted in a near-axial dissection [13]. As such, tangential sectioning cannot be entirely avoided, but fortunately, it renders DOI measurement difficult in only a minority of cases (10%) in our hands. Higher proportions (55%) reported by others (Aoyama) may be due to slicing at thicker intervals (5–7 mm vs. 3 mm according to our grossing protocol), which reduces the number of specimen slices and consequently, the chance of obtaining slices with proper cross-sectioning of the CBD. A further, minor cause for confusion among pathologists is the point from which DOI should be measured. Some authors [9, 10] advocate measuring from the basal membrane of the bile duct epithelium, whereas others have noted that this structure is often fragmented [13]. In practice, this is largely a moot point, as the nonneoplastic/dysplastic epithelial lining usually is absent, either due to cancer infiltration or stent-related ulceration. Thus, in most cases, the practical approach is to measure DOI from the luminal surface of the tumour (Figure 1).

The R1-rate in this study (71.8%) is high in comparison to previously reported rates, which vary from 1.8% to 50.0% [10–13, 18–20]. A likely reason for the substantially higher R1-rate is that – in contrast to other studies – the circumferential margin (also known as the radial margin) of the extrapancreatic bile duct was included in the evaluation and found to be frequently involved (25.4%) in our study. In addition, axial slicing and extensive en bloc sampling of the tumour onto the nearest specimen surfaces also likely contributed to a higher detection rate of microscopic margin involvement. Similarly, high accuracy of the pathology examination procedure is also the likely reason for the higher rate of lymph node metastasis (78.9% N+) than in other studies (31.5% Hong 2007, 33.5% Jun 2019, 40.6% Aoyama 2018, 63.3% Hong 2009).

This study has several limitations. Firstly, it is a retrospective, single-centre study, which may limit the generalisability of our findings. Secondly, the relatively small sample size affects the statistical power of certain analyses. Finally, variations in postoperative treatments, such as chemotherapy, were not accounted for and may have influenced survival outcomes.

## Conclusion

In summary, our study confirms in a single-centre Western cohort that the 8^th^ edition of the UICC/AJCC TNM classification provides superior prognostic stratification for distal CCA compared to the 7^th^ edition by using DOI as a criterion for T-stage classification and introducing a 3-tiered N-classification. These refinements improve risk stratification, but rely on meticulous pathology assessment, in particular accurate measurement of DOI and thorough examination of lymph nodes and margins, the latter two variables being independent predictors of poor survival.

## Supplementary Material



## Data Availability

The data that support the findings of this study are not publicly available, but are available from the corresponding author upon reasonable request.
