# Supplementary material for: Outcome of resectable distal cholangiocarcinoma in a single-centre Western patient cohort: comparison of the 7th and 8th edition of the UICC/AJCC TNM classification
Source: Acta Oncol. 2026 Feb 19;65:44965. doi: 10.2340/1651-226X.2026.44965 (PMC12931082; doi:10.2340/1651-226X.2026.44965)
Supplement: Supplementary file 1 [file AO-65-44965-s1.pdf]

Supplementary material has been published as submitted. It has not been copyedited, or typeset by Acta Oncologica

Supplementary material

Table S1. Uni- and multivariable Cox regression analyses of prognostic factors for survival in patients undergoing pancreatoduodenectomy for distal bile duct cancer.

**Parameters**	**Univariable**		**Multivariable†**	
	**HR (95% CI)**	**p-value**	**HR (95% CI)**	**p-value**
Age, years	1.03 (0.99-1.07)	0.16		
Gender (male)	0.72 (0.42-1.23)	0.23		
Body mass index, kg/m^2	1.04 (0.96-1.12)	0.34		
Presence of comorbidity	1.63 (0.77-3.47)	0.2		
Hypertension	0.89 (0.52-1.53)	0.69		
Cardio-vascular disease	0.76 (0.41-1.43)	0.4		
ASA score III (vs II)	0.97 (0.57-1.65)	0.91		
Preoperative CA 19-9 level	1.001 (1.000-1.002)	0.09		
Intrapancreatic tumour location (vs extra-/intra- + extrapancreatic location)	0.65 (0.37-1.14)	0.13		
Tumour size, mm	1.05 (1.01-1.09)	0.013	________________	-
pT3 stage (vs pT2) 8th edition	1.86 (1.02-3.39)	0.044	________________	-
Lymph node yield	0.98 (0.94-1.03)	0.37		
Lymph node ratio	1.02 (1.004-1.03)	0.015	________________	-
pN stage (vs pN0)				
pN1	1.92 (0.82-4.56)	0.13	________________	-
pN2	2.59 (1.11-6.07)	0.028	________________	-
TNM stage (7th edition)				
I B/II A	baseline			
II B	2.23 (0.99-4.96)	0.051		
TNM stage (8th edition)				
I/II A	baseline			
II B	2.25 (0.91-5.58)	0.063	________________	-
III A	2.94 (1.19-7.26)	0.019	________________	-
Poor differentiation (vs well/moderate)	1.36 (0.77-2.4)	0.28		
Vascular invasion	2.11 (1.21-3.68)	0.028	1.84 (1.02-3.29)	0.042
R1 margin status	2.85 (1.43-5.72)	0.003	3.08 (1.37-6.92)	0.007
Adjuvant chemotherapy	0.59 (0.32-1.08)	0.09		

† *Backward regression analysis*

Figure S1. Survival according to T stage (7th and 8th edition)†.

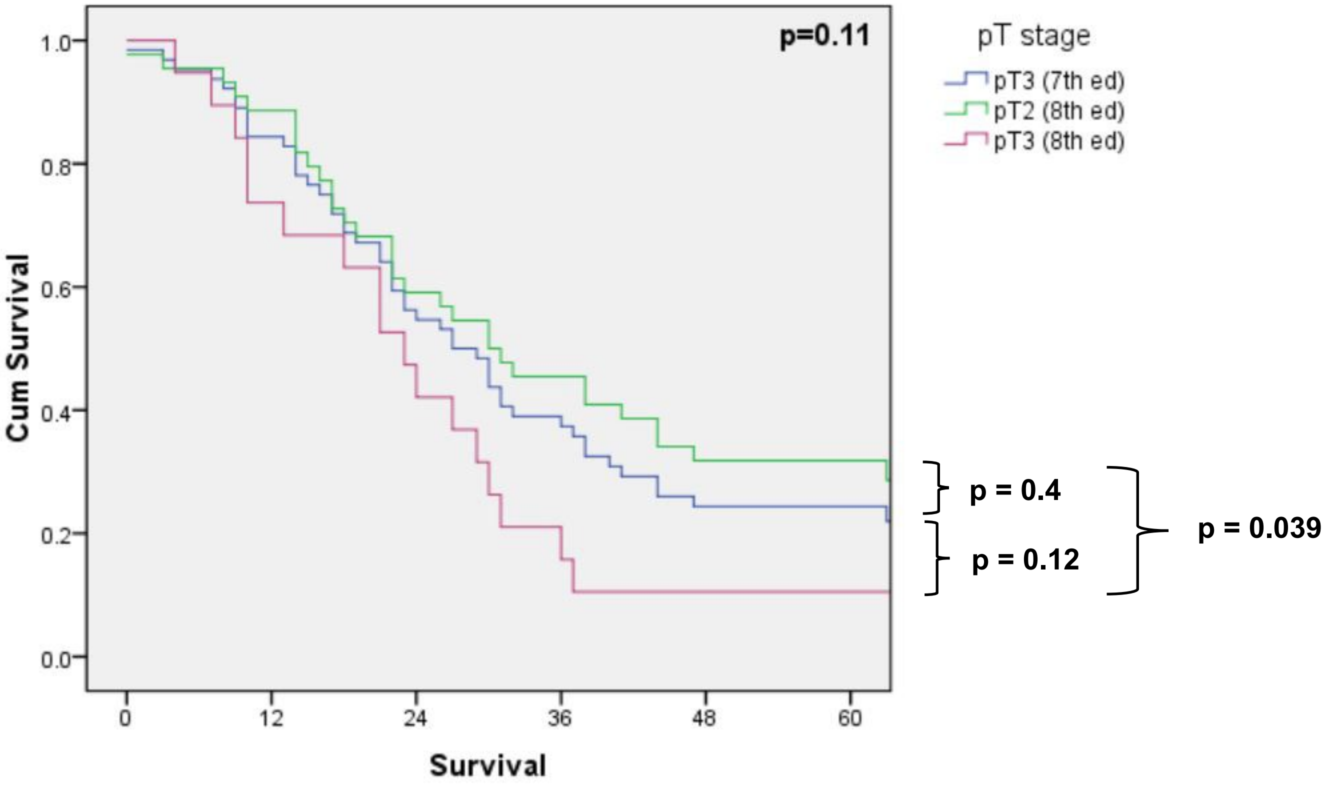

Patients at risk*						
pT3 (7th ed)	64	54	36	24	15	11
pT2 (8th ed)	44	39	26	20	14	11
pT3 (8th ed)	19	14	9	4	2	1

Parameter	Survival				p-value		
	Median (95% CI), months	1-year	3-year	5-year			
pT3 (7th ed)	27 (20.1 - 33.9)	84.4%	37.4%	24.4%		0.4	0.12
pT2 (8th ed)	30 (18.9 – 41.1)	88.6%	45.5%	31.8%	0.4		0.039
pT3 (8th ed)	23 (16.6 – 29.4)	73.7%	15.8%	10.5%	0.12	0.039	

†*Those with pT2 (7th ed) and pT1 (8th ed) stage distal bile duct cancer was excluded due to the small numbers – 4 patients in each group.*

**Four patients who had died within 90 days of surgery due to postoperative complications were excluded from survival analysis.*

Figure S2. Survival according to N stage (8th edition).

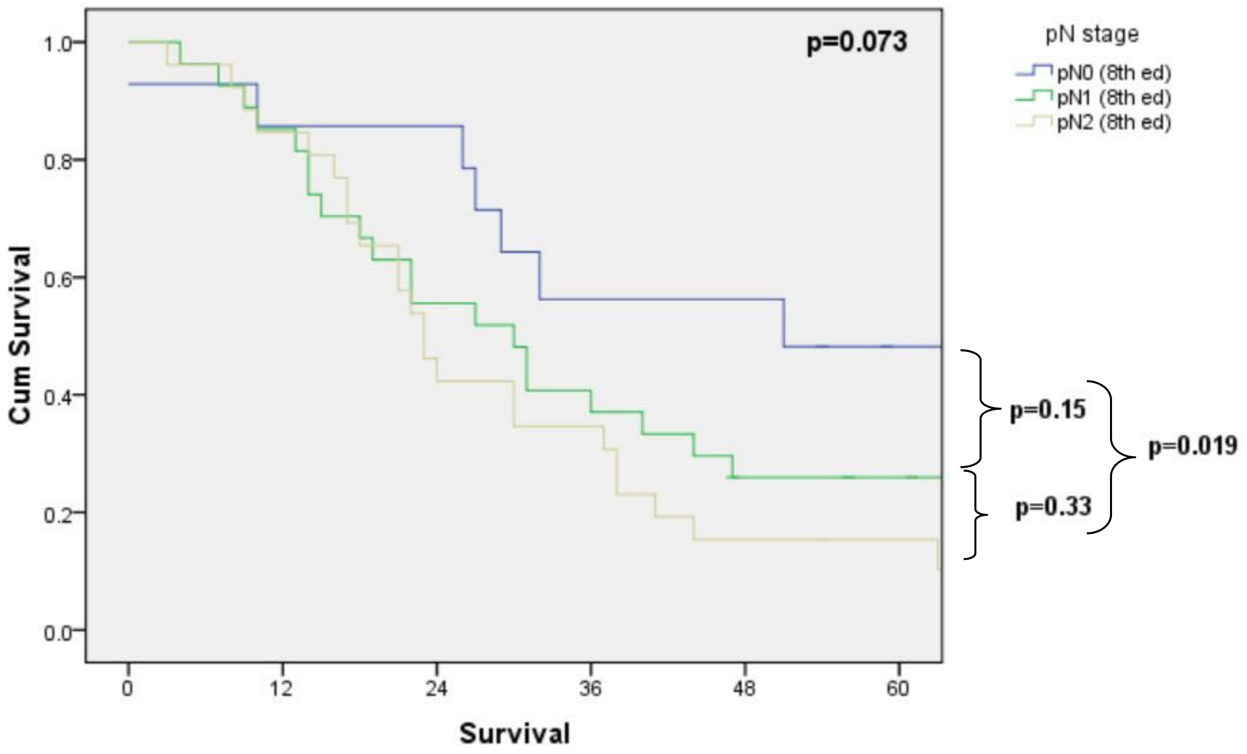

**Patients at risk**						
pN0	14	12	12	7	7	4
pN1	27	23	15	11	6	5
pN2	26	22	12	9	4	3

**Parameter**	**Survival**				**p-value**		
	**Median (95% CI), months**	**1-year**	**3-year**	**5-year**			
pN0	51 (…)	85.7%	56.3%	48.2%		0.15	0.019
pN1	30 (18.6 – 41.5)	85.2%	37%	25.9%	0.15		0.33
pN2	23 (19.3 – 26.7)	84.6%	34.6%	15.4%	0.019	0.33	

Figure S3. Survival according to TNM stage (7th and 8th edition)†.

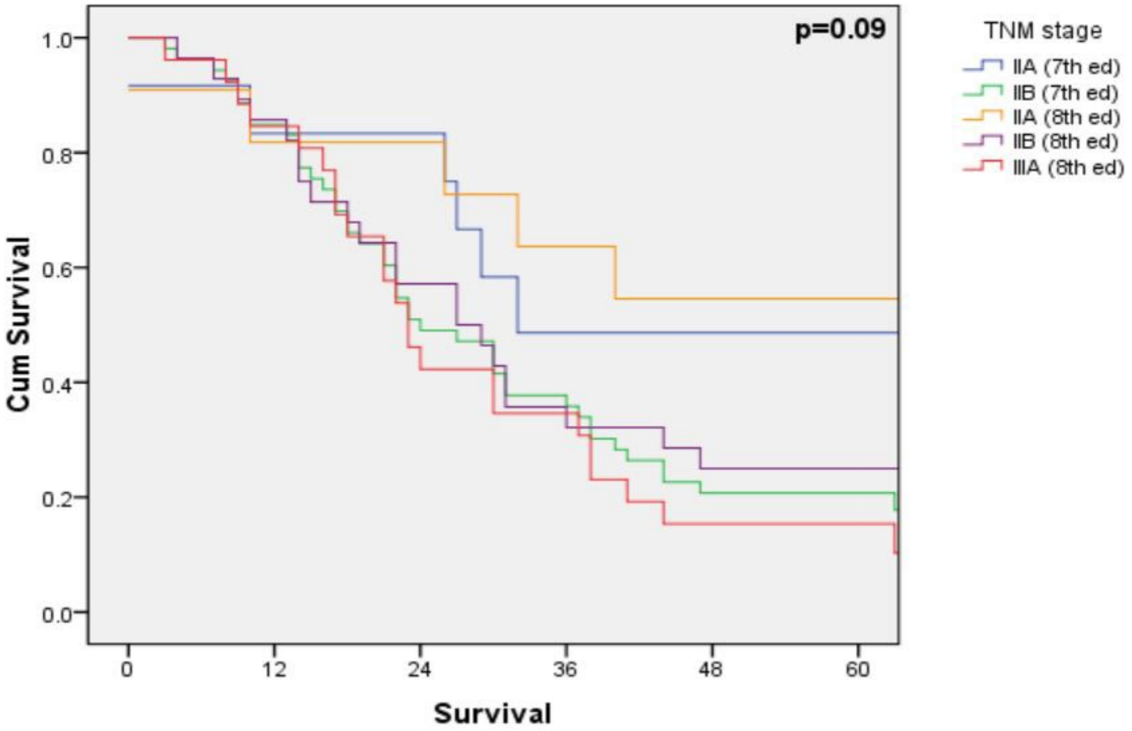

**Patients at risk**						
IIA (7th ed)	12	10	10	5	5	3
IIB (7th ed)	53	45	27	20	10	8
IIA (8th ed)	11	9	9	7	5	3
IIB (8th ed)	28	24	16	10	7	6
IIIA (8th ed)	26	22	12	9	4	3

**Parameter**	**Survival**				**p-value**				
	**Median (95% CI), months**	**1-year**	**3-year**	**5-year**					
IIA (7th ed)	32 (….)	83.3%	48.6%	48.6%		0.092	0.73	0.2	0.048
IIB (7th ed)	24 (17.5 - 30.4)	84.9%	35.8%	20.8%	0.092		0.042	0.66	0.57
IIA (8th ed)	__________	81.8%	63.6%	54.5%	0.73	0.042		0.11	0.021
IIB (8th ed)	27 (16.6 - 37.3)	85.7%	32.1%	25%	0.2	0.66	0.11		0.38
IIIA (8th ed)	23 (19.3 – 26.7)	84.6%	34.6%	15.4%	0.048	0.57	0.021	0.38	

† *Patients with TNM stage 1b (7th edition) and 1 (8th edition) distal bile duct cancer were excluded due to small numbers - 3 and 2 patients, respectively.*

Figure S4. Survival according to tumour location in the distal bile duct.

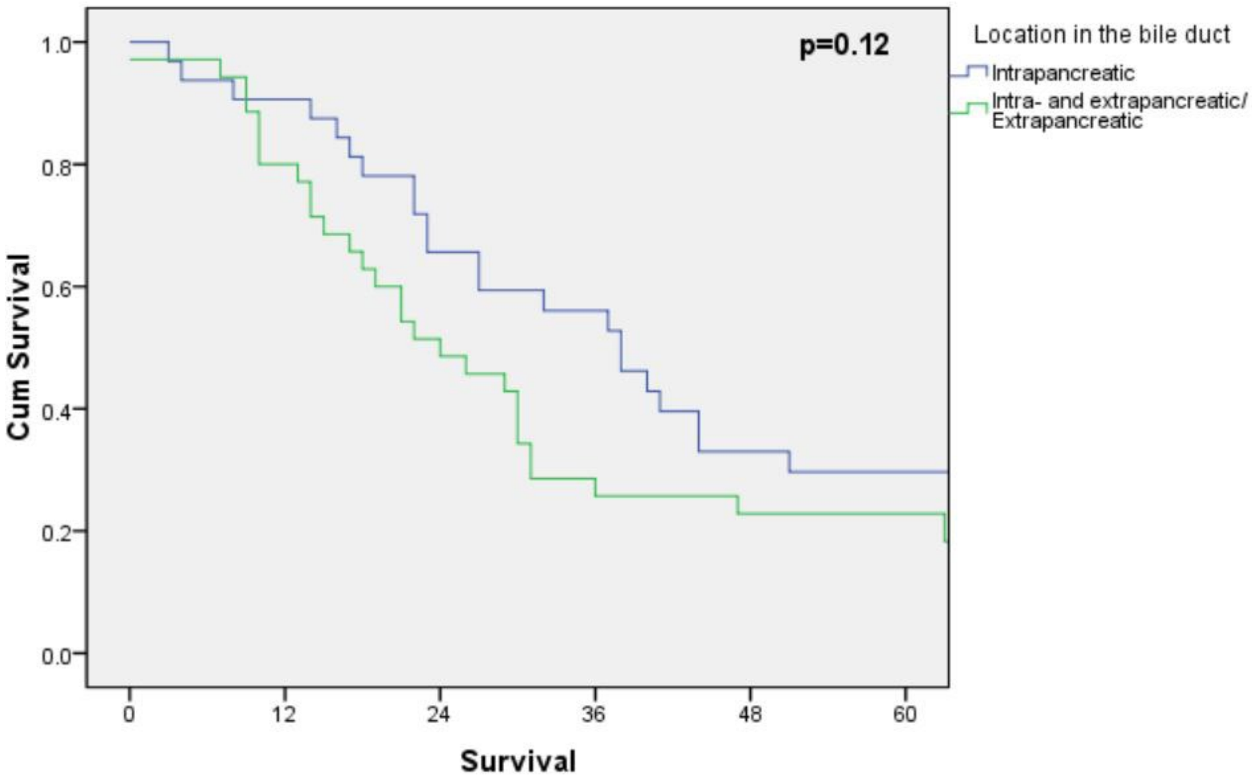

Patients at risk						
Intrapancreatic	32	29	21	17	10	7
Intra+extrapancreatic/ Extrapancreatic	35	28	18	10	7	5

Parameter	Survival				p-value
	Median (95% CI), months	1-year	3-year	5-year	
Intrapancreatic	38 (27.4 - 48.6)	90.6%	56.1%	29.7%	0.12
Intra+extra/ extrapancreatic	24 (14.3 - 33.7)	80%	25.7%	22.9%	
